# Mutagenesis of α-Conotoxins for Enhancing Activity and Selectivity for Nicotinic Acetylcholine Receptors

**DOI:** 10.3390/toxins11020113

**Published:** 2019-02-13

**Authors:** Matthew W. Turner, Leanna A. Marquart, Paul D. Phillips, Owen M. McDougal

**Affiliations:** 1 Biomolecular Sciences Graduate Programs, Boise State University; Boise, ID 83725, USA; matthewturner1@u.boisestate.edu; 2 Department of Chemistry and Biochemistry, Boise State University; Boise, ID 83725, USA; leannabrown@boisestate.edu (L.A.M.); paul.dan.phillips@gmail.com (P.D.P.)

**Keywords:** α-conotoxins (α-CTxs), nicotinic acetylcholine receptors (nAChRs), mutational analysis, positional scanning synthetic combinatorial libraries (PS-SCL), acetylcholine binding protein (AChBP), protein surface topography (PST), genetic algorithm managed peptide mutant screening (GAMPMS), molecular dynamics (MD), solid phase peptide synthesis (SPPS)

## Abstract

Nicotinic acetylcholine receptors (nAChRs) are found throughout the mammalian body and have been studied extensively because of their implication in a myriad of diseases. α-Conotoxins (α-CTxs) are peptide neurotoxins found in the venom of marine snails of genus *Conus*. α-CTxs are potent and selective antagonists for a variety of nAChR isoforms. Over the past 40 years, α-CTxs have proven to be valuable molecular probes capable of differentiating between closely related nAChR subtypes and have contributed greatly to understanding the physiological role of nAChRs in the mammalian nervous system. Here, we review the amino acid composition and structure of several α-CTxs that selectively target nAChR isoforms and explore strategies and outcomes for introducing mutations in native α-CTxs to direct selectivity and enhance binding affinity for specific nAChRs. This review will focus on structure-activity relationship studies involving native α-CTxs that have been rationally mutated and molecular interactions that underlie binding between ligand and nAChR isoform.

## 1. Introduction

Snails of the genus *Conus* inhabit tropical and subtropical waters, where they hunt prey by injection of a venom rich in pharmacologically active peptides, referred to as conopeptides [1,2,3]. Cone snails are relatively slow moving aquatic organisms and have evolved venom to serve as an efficient chemical cocktail for prey incapacitation, retrieval and predator protection. The diversity of conopeptides is staggering. With >700 known *Conus* species, the venom of each species is comprised of hundreds to thousands of distinct conopeptides, equating to hundreds of thousands of unique, pharmacologically active conopeptides [4,5,6]. As of December 2018, the database for conopeptides, ConoServer, contained 6274 entries for conopeptide proteins with 220 protein structures [7].

Conotoxins (CTxs) are a subset of conopeptides that are small, disulfide rich peptides which primarily target voltage or ligand gated ion channels [8,9]. CTxs are classified by their molecular targets, as indicated by the first letter in Greek in the naming convention based on the International Union of Basic and Clinical Pharmacology Committee on Receptor Nomenclature and Drug Classification system [10,11]. For example, ω-CTxs target voltage gated calcium channels, δ- and μ-CTXs target voltage gated sodium channels, χ-CTxs target norepinephrine transporter, ρ-CTxs target α1A-adrenoreceptor, and α-CTxs target nicotinic acetylcholine receptors (nAChRs) [11,12]. Conopeptides are also classified into 16 genetically distinct superfamilies, to which α-CTxs primarily belong to the A and O superfamilies [11]. This diversity in molecular targets make CTxs particularly useful research tools for understanding the physiological role of indigenous mammalian receptors and ion channels, thereby making CTxs appealing as prospective selective therapeutics for human diseases. The therapeutic potential of CTxs is illustrated in the example of ω-CTx MVIIA. ω-CTxs have been investigated as therapeutic drugs due to their ability to selectively bind voltage-gated calcium channels (VGCCs), which are directly associated with pain pathways [13]. ω-CTx MVIIA, marketed by Elan Corporation under the trade name PrialtⓇ, is a nonaddictive pain analgesic, 1000 times more potent than morphine [14]. Following twenty years of research, the United States Food and Drug Administration accepted the use of PrialtⓇ to treat chronic pain in 2004 [15].

The focus of this review is α-CTxs, which target and act as competitive antagonists of nAChRs. α-CTxs generally contain between 12–20 amino acid residues, including four cysteines that form two highly conserved disulfide bonds. In native α-CTxs, the disulfide bonds are formed such that Cys1 binds Cys3, and Cys2 binds Cys4. The first and second cysteine residues are always adjacent, but the number of amino acid residues between Cys2 and Cys3, and between Cys3 and Cys4 can vary, resulting in two loops of intervening amino acids denoted *m* and *n*, respectively. The cysteine framework refers to the number of residues in the *m* and *n* loops. For example, α-CTxs with a 4/7 cysteine framework contain four and seven residues in their respective *m* and *n* loops. 

nAChRs are a class of ligand-gated ion channels in the Cys-loop superfamily, to which γ-aminobutyric acid (GABA), glycine and 5-HT_3_ receptors also belong [16,17]. nAChRs are pentameric ligand-gated ion channels found in both the central and peripheral nervous systems in mammals [18]. To date, 16 human nAChR subunit genes coding for subunits α1–α7, α9, α10, β1-β4, γ, δ, and ε have been identified [19]. nAChR subunit expression varies by tissue, with α1, β1, γ, δ, and ϵ subtypes being expressed in muscle. α2–α7, α9, α10, and β2–β4 subunits are referred to as “neuronal,” despite their presence in non-neuronal tissues [18,20]. nAChRs can be composed of heterogeneous or homogenous combinations nAChR subunits, forming different nAChR isoforms, with distinct physiological properties. Heteromeric combinations of α2–α6 and β2–β4, complexes of α9α10, and homomeric combinations of α7 or α9 are known to exist [21]. nAChR subunits are composed of an N-terminal extracellular domain that contains the acetylcholine binding site, four hydrophobic transmembrane domains that form the ion-pore and an intracellular loop [22,23,24]. 

nAChRs are pharmaceutically important because they modulate the release of neurotransmitters (e.g., glutamate, norepinephrine, dopamine, acetylcholine), and because particular nAChR isoforms are expressed within specific neuronal pathways [25]. Different nAChR subunits may be expressed in defined regions in the mammalian body [25]. For example, dopaminergic neurons of the midbrain express α3–α7 and β2–β3 subunits, resulting in expression of nAChR isoforms, such as α4α6β2β3, α4α6β2 and α6β2β3 that are highly and specifically expressed in select neuronal pathways [26,27]. Dopaminergic pathways are involved in numerous processes, including reward/reinforcement, attention, cognition, and voluntary movement. The well-defined and specific expression of α6-containing nAChRs in these neuronal types suggests they may be candidate drug targets for more selective approaches to treating disorders involving dopamine transmission [28,29,30].

The significance of ligands that selectively bind to nAChRs lies in the variety and severity of neurological disorders in which degradation or altered activity of nAChRs is associated. Examples of neurological disorders associated with nAChRs are schizophrenia, nicotine addiction, Alzheimer’s disease and Parkinson’s disease [28,31,32,33]. Therefore, differentiating between nAChR isoforms is an important step in better understanding neurological disorders [34,35]. The high degree of sequence homology between different nAChR subunits makes discovery of selective antagonists for particular nAChR isoforms challenging. The lack of selectivity for the intended channel subtype could cause serious side effects and represents a major hurdle in the application of α-CTxs in pharmacological context. Frequently, a particular α-CTx is capable of inhibiting multiple nAChR isoforms [36]. For example, α-CTx MII blocks both α3β2 nAChR and α6-containing nAChRs isoforms, α-CTx ImI blocks α3β2, α7, and α9 nAChR subtypes, and α-CTx OmIA block α7, α3β2, and α6/α3β2β3 nAChRs. Although it may be evolutionarily advantageous for CTxs to be promiscuous amongst related receptors, this is undesirable in pharmaceutical and biotechnological applications. 

For this reason, much effort has gone into the mutation of native CTxs to enhance their selectivity and potency to specifically target individual nAChR subtypes. Mutagenesis can also provide information regarding the significance of a given amino acid in the potency and selectivity of a CTx, as well as provide information regarding the molecular determinants that govern ligand/receptor interactions. The purpose of this review is to explore strategies researchers have employed to mutate CTxs for improved selectivity and potency and describe—when possible—the structure-function relationships that govern the ligand-receptor interaction. Mutations introduced to synthetic α-CTxs to improve their pharmacological properties have been reviewed previously [37]. However, recent insights into the molecular structure of nAChRs and the development of contemporary computational tools capable of rational mutagenesis of α-CTxs merit an additional review of this topic. Table 1 includes the primary sequence and the activity of the CTxs discussed in this review. Recent excellent reviews have summarized the activity of native CTxs in a more comprehensive manner [11,13,36,37,38,39,40]. We start with a general discussion of different strategies used to rationalize and inform α-CTx analogue development and then provide a brief review of structural data available for nAChRs, followed by specific structure activity relationship examples of the mutational analysis of individual α-CTxs designed to target specific nAChR isoforms. 

## 2. Mutagenesis Strategies for α-Conotoxins

Various approaches have been employed to alter the primary sequence of α-CTxs to enhance their affinity and selectivity for particular nAChR subtypes. Alanine scanning mutagenesis has been used extensively to determine the importance of a given amino acid in binding of a particular α-CTx to a particular receptor. Changes in activity for a mutated amino acid provide information on the significance of a given residue in the binding event and provide insight to the interaction between the α-CTx and the corresponding structural features in the nAChR binding pocket. Systematic replacement of residues with non-Ala amino acids also provides more subtle detail regarding the ligand/receptor interactions of CTxs with nAChRs. Most mutagenesis studies of α-CTxs have focused on single amino acid substitutions, with a more limited number of reports describing synthesis and analysis of α-CTx analogs. Recent approaches have applied mixture-based combinatorial methods as a high throughput strategy to synthesize α-CTx analogs. The use of positional scan synthetic combinatorial libraries (PS-SCLs) has provided a way of acquiring functional information regarding all possible variable positions within the α-CTx framework. This approach relies upon medium to high throughput screening assays for particular nAChR receptor subtypes, and screening of pooled mixtures of α-CTx analogs to determine which amino acid positions confer higher activity. PS-SCL allows for the determination of a consensus sequence, which is an α-CTx peptide with multiple site mutations, intended to exhibit greater affinity for the desired nAChR subtype. The consensus sequence peptide can then be synthesized for focused pharmacological analysis using electrophysiology recordings. 

Computational modeling of α-CTx/nAChR interactions have emerged as a useful tool for rationalizing mutagenesis design. In particular, the use of computational docking methods coupled with molecular dynamics (MD) simulations has proven useful for accelerating the discovery α-CTx analogues with enhanced binding affinity and selectivity for target nAChRs. In this methodology, the most energetically favorable binding poses predicted by computational docking are used as a starting point for MD simulations. The affinity of the α-CTx/nAChR interactions can be increased by inspecting the ligand/receptor binding paradigm and mutating those residues on the peptide that could lead to more, or stronger, contacts with the residues in the receptor’s binding site. Because such structure-based virtual screening protocols depend upon structural data for nAChRs on the atomic scale, we will briefly review the advances in the structural data available for nAChRs and the application of these data to generated models for particular nAChR subtypes.

## 3. nAChR Structural Data to Rationalize α-CTx Mutagenesis

**Figure 1:**
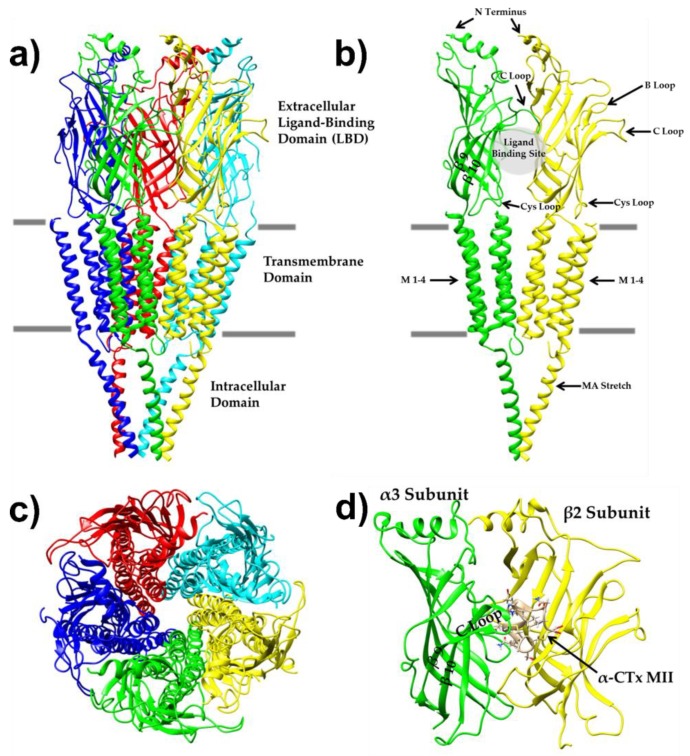
The general structure of the nAChR, from the crystal structure of the *Torpedo marmorata* (PDB: 2BG9). (**a**) View from the side of the nAChR, parallel with the cell membrane, showing the extracellular, transmembrane, and intracellular domains. (**b**) Dimer of two subunits showing the ligand binding site and select loops and helical regions. (**c**) Top view of the pentametic nAChR looking through the conducting pore. (**d**) A homology model of the ligand-binding domain (LBD) of an α3 (shown in green) and β2 (shown in yellow) dimer with α-CTx MII bound, illustrating the orientation of CTx binding in context of the structural features of the nAChR subunits. Figures were prepared using UCSF Chimera [63].

Electron microscopic analysis of nAChRs isolated from the electric ray organ of *Torpedo marmorata* provided the first structural insights to human nAChRs [41]. This study revealed the overall organization of the pentameric assembly and the architecture of the extracellular domain and the pore region of nAChRs. However, in-depth information about the fine molecular details of the ligand binding domain was limited by the low resolution at 4 Å [42]. The general structure of the nAChR is shown from the side, in parallel with the cell membrane in Figure 1a, and through the ion pore perpendicular to the cell membrane in Figure 1c. Another breakthrough in the understanding of the atomic structure of nAChRs was the discovery of acetylcholine binding protein (AChBP), a non-channel homolog of the extracellular domain of nAChRs, first isolated and crystalized from the fresh water snail *Lymnea stagnalis* (*Ls*) [43]. Other AChBPs were subsequently identified in *Bulinus truncatus* (*Bt*) and *Aplysia californica* (*Ac*) [44,45]. Many crystal structures of AChBPs have been solved in different ligand-bound states, providing atomic-resolution details of the interactions between the extracellular domain and a variety of agonists and antagonists [46], including α-CTx PnIA [45] and cobratoxin [47]. Combined, these studies provided a wealth of structural information upon which homology models for human nAChRs have been constructed. However, AChBPs do not function as ion channels and may lack the necessary structural features required for ligand-binding signals across the protein body. Additionally, AChBP shares only 20–25% sequence homology with the nAChRs, further limiting their utility as structural templates for homology models of nAChRs. More recently, the x-ray structure of the extracellular domain of the mouse α1 nAChR was solved, revealing two hydrophilic residues conserved in nAChRs (and not AChBP) that are buried in the internal portion of the protein [48]. These observations strongly suggest that the hydrophilic interior of nAChRs is important for channel function, probably by conferring the receptor-specific structural flexibility required for the allosteric channel gating. Additionally, the crystal structures of homopentamers of human α7 [49] and α9 [50] nAChRs were solved. The X-ray crystallographic structure of the human α4β2 nAChR with nicotine bound was recently solved [51]. Several studies have used the 3D structure of AChBP as a template for homology model creation of nAChRs [52,53], and a few examples exist where the mouse α1 subunit was used as a template [54,55]. Recently, studies have used the crystal structure of the homomeric human α9 nAChR subtype [56,57], as well as the crystal structure of the heteromeric human α4β2 nAChR subtype [58] to inform the homology models of heteromeric nAChR subtypes. However, because effective virtual screening requires that the conformation of the ligand bound complex be known to high accuracy, AChBPs still represent the most commonly used template for the characterization of α-CTx binding to the extracellular ligand-binding domain of nAChRs [59,60,61,62]. 

## 4. α-Conotoxin Mutational Analysis

### 4.1. α-CTx Vc1.1

α-CTx Vc1.1, with primary sequence GCCSDPRCNYDHPEIC, was first discovered using PCR screening of cDNAs isolated from the venom ducts of *Conus victoriae* in 2003 [64]. Later, this α-CTx peptide was identified in the venom of *C. vicoriae* using mass spectrometry, and found to contain an amidated C-terminus, as well as post-translationally modified hydroxyproline and γ-carboxyglutamate in positions Pro6 and Glu14, respectively [65]. The native, post-translationally modified peptide was designated vc1a to distinguish it from the synthetic Vc1.1 containing the same primary sequence. Synthetic Vc1.1 was shown to antagonize neuronal nAChRs in bovine chromaffin cells and was demonstrated to alleviate neuropathic pain in rat models of human neuropathic pain, as well as accelerate the functional recovery of injured neurons [66]. Clark et al. determined the NMR solution structure of α-CTx Vc1.1 and characterized the inhibition of individual nAChRs using electrophysiology with *Xenopus* oocytes expressing various rat nAChR subtypes [67]. α-CTx Vc1.1 demonstrated inhibition of α3α5β2, α3β2, and α3β4 nAChRS, with IC_50_ values of 7.2, 7.3, and 4.2 μM, respectively. No inhibition was demonstrated for α3α5β4, α4β2, α4β4, α7, or αβγδ nAChRs up to >30 μM. Although it was initially thought that α-CTx Vc1.1 targets α3-containing nAChR, subsequent reports demonstrated a much higher affinity for the α9α10 nAChR subtype, suggesting this may be the basis for the reduction in neuropathic pain upon α-CTx Vc1.1 administration [68,69].

A scanning mutagenesis study in 2009 by Halai et al. aimed to determine the key residues and interactions that govern the binding of α-CTx Vc1.1 to the α9α10 nAChR subtype, because of this receptor’s potential as a therapeutic target for the treatment of neuropathic pain [70]. It was determined the IC_50_ of Vc1.1 for rα9α10, hα9α10, and rα3β2 nAChR isoforms at 0.109, 0.549, and 5.532 μM, respectively. They performed a two-stage mutation screen on each non-Cys residue to either an “inert” residue (Ala), a negatively charged residue (Asp), or a positively charged residue (Lys) to identify key amino acids in the interaction of Vc1.1 with the r/hα9α10 nAChR isoforms. All mutations imposed on Vc1.1 showed clear selectivity for r/hα9α10 over rα3β2. They first identified Asp5–Arg7 and Asp11–Ile15 as the key residues essential to maintain functional activity. When Asn9 was replaced with a hydrophobic Ala, Leu, or Ile, the IC_50_ was reduced for rα9α10, hα9α10, and rα3β2 nAChR isoforms from 109, 549, and 5532 nM to 13, 27, 185 nM, respectively. Mutation of Ser4 with a positive residue, either Lys or Arg, shifted the selectivity of Vc1.1 from rα9α10 to hα9α10 and lowered the IC_50_ for hα9α10 to 19 nM. Ser4 is highly conserved among many α-CTxs (see Table 1), and a smaller surface hydrophobic patch in loop 1 of 4/7 α-CTxs is thought to increase specificity for rα3β2. 

In an electrostatic map of Vc1.1, when Asn9 was replaced with a hydrophobic residue (Ala), the two smaller hydrophobic patches on opposite ends of the α-helix merged into a larger hydrophobic surface across one side of the α-helix. The result of the N9A mutation to Vc1.1 was a 30-fold increase in potency to inhibit the function of rα3β2 and roughly a 10-fold increase in inhibition of r/hα9α10, suggesting that extending the hydrophobic patch on this side of the peptide is favorable for binding to rα3β2 as well as r/hα9α10.

### 4.2. α-CTx PeIA

α-CTx PeIA was originally cloned from a cDNA library of *Conus pergrandis* and has the primary sequence GCCSHPACSVNHPELC [91]. α-CTx PeIA demonstrated inhibition of rα9α10, α6/α3β2β3, α3β2, and α3β4 with IC_50_ values of 6.9, 17.2, 19.2, and 480 nM, respectively [71]. The sequence of PeIA has a proline residue in position 6, which is consistent with α-CTxs MII and GIC [83,92]. Pro6 initiates the α-helix in PeIA in the same way as is observed for MII and GIC, indicating that the peptide backbone of PeIA may have a similar molecular scaffold. In addition, PeIA has a critical His12 residue, consistent with His12 in MII and similar to Asn12 in GIC. Consequently, these three peptides show comparable activity with α3β2 nAChR isoforms: PeIA and MII inhibit the rα3β2 nAChR isoform with IC_50_ values of 9.7 and 0.5 nM respectively, and GIC inhibits the hα3β2 nAChR isoform with an IC_50_ of 1.13 nM [83,92]. Unlike MII or GIC, PeIA also inhibits rα9α10 with an IC_50_ of 6.9 nM. Thus, the amino acids unique to PeIA (S8, V9, N10, P13, and E14) are likely the determinants of its high selectivity for the rα9α10 nAChR isoform.

α-CTx LvIA, GCCSHPACNVDHPEIC, has the same composition of amino acids in loop 1 as PeIA, but differs by three amino acids (LvIA:PeIA, N9S, D11N, and I15L) in loop 2 [55]. The IC_50_ values of PeIA and LvIA for rα3β2 were determined to be 9.7 and 8.65 nM, respectively. LvIA also has high divergence to other α-CTxs in loop 2 and is the first report of an α-CTx with a strong rα3β2 selectivity over rα6/α3β3β2. Both PeIA and LvIA have similar residues at positions 7 and 10, but PeIA inhibits rα9α10, while LvIA does not. Luo et al. propose that this discrepancy could be explained by PeIA binding at a different site on the rα9α10 nAChR isoform than LvIA. The rα9α10 nAChR isoform has two α-CTx binding sites, one at the α9α10 subunit interface and another at the α10α9 subunit interface. The α10α9 subunit interface displays more charged residues than the α9α10 subunit interface. For example, four charged side chains of Vc1.1 (R7, D11, H12, and E14) would be expected to have electrostatically favorable binding to the α10α9 subunit interface. By contrast, PeIA has only one charged side chain (Glu14) in loop 2 and would be expected to bind to the more hydrophobic binding site at the α9α10 subunit interface. The observation that LvIA does not inhibit rα9α10 containing nAChRs may be explained by the fact that LvIA has charged side chains at Glu14 and Asp11 that could reduce affinity for the α9α10 interface, and binding to the α10α9 interface is unlikely due to poor shape complementarity to the binding site.

Yu et al. provided insight into PeIA binding with rα9α10 using a computational homology model generated to describe the binding paradigms for Vc1.1 based on the crystal structure of *Ac*-AChBP in complex with PnIA [A10L, D14K] (PDB: 2BR8) [45] and the crystal structure of hα9 (PDB: 4UY2) [55]. Similar to Vc1.1, PeIA residues H12 and E14 interact with residues E197 of α10(+) and R113 of α9(−), respectively. However, in contrast to Vc1.1, PeIA does not interact with N154 of α9(+) because the side chain of N154 is not in close proximity to PeIA, and PeIA has an additional hydrogen bond between residue N11 (D11 in Vc1.1) and T152 in both α10(+) and α9(+). This suggests PeIA has a lack of binding site preference between α10(+)-α9(−) and α9(+)-α9(−). Indeed, PeIA showed no stoichiometric-dependent activity with hα9α10, suggesting it has no preferred binding to α9(+)-α9(−) versus α10(+)-α9(−).

α-CTx PeIA was used as a starting point to conduct a positional scanning mutagenesis study by Hone et al. to develop a ligand that selectively bound α6β2β3 vs α3β2 [72]. Substitution of His5 and His12 with alanine, and Pro6 with hydroxyproline dramatically reduced potency for both receptor types. Alanine substitution with Glu14 and Leu15 modestly decreased affinity for α3β2, and substitution of Asn11 with alanine, and Pro13 with hydroxyproline had little effect on the potency at either receptor isoform. In summary, substitution of non-cysteine residues with alanine had no significant impact on the ability of α-CTx PeIA to discriminate between α6β2β3 and α3β2. 

This led the authors to use amino acid substitutions based on the primary sequence of other α-CTxs, including α-CTx MII and α-CTx TxIB, to guide the development of a peptide capable of discriminating between these closely related nAChR subtypes. Based on the sequence of α-CTx MII, H5N, A7V, V10L, N11E, and P13S were analyzed. Of these, the A7V mutation increased the potency for α6β2β3 while decreasing potency for α3β2, resulting in a ~12-fold enhanced selectivity for α6β2β3. α-CTx TxIB is exclusively selective for α6β2β3 [81] and differs from α-CTx PeIA by only six residues, leading to several interesting single mutations. The α-CTx PeIA [S9R] increased potency for both subtypes, whereas α-CTx PeIA [V10R] significantly reduced potency at both subtypes. The N11R substitution eliminated activity at α3β2, while minimally affecting the potency at α6β2β3. Combining these novel mutations that preferentially bind α6β2β3 over α3β2 with [S9H, V10A] analog previously identified with enhanced potency against both α6β2β3 and α3β2 resulted in the combined substitution mutant α-CTx PeIA [A7V, S9H, V10A, N11R, E14A]. This mutant resulted in a >15,000-fold higher affinity for α6β2β3 over α3β2 and enhanced potency at α6β2β3 (IC_50_ 2.16 nM). 

**Figure 2:**
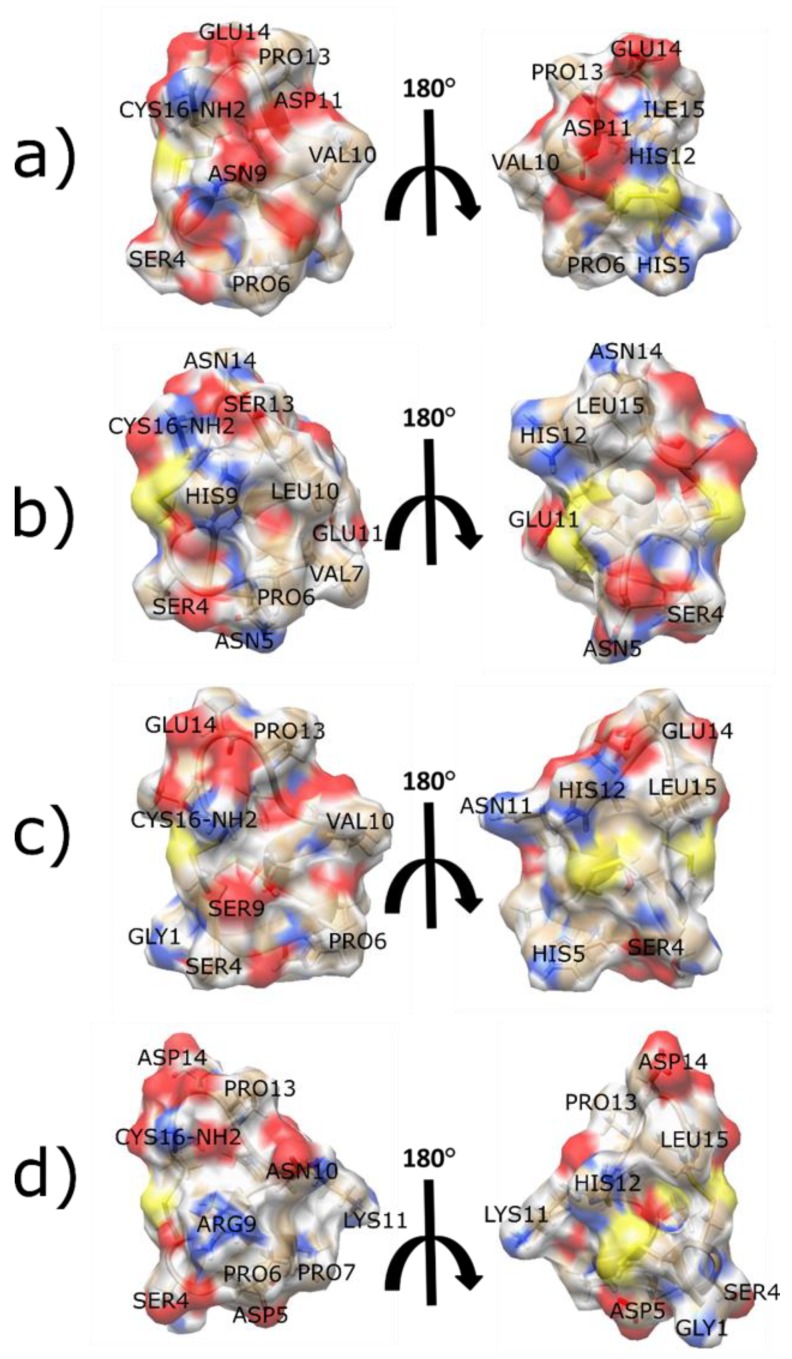
Structural comparison of select α-CTxs (**a**) LvIA (**b**) MII (**c**) PeIA and (**d**) TxIB. These bind α3β2- and/or α6α3β2β2-nAChR. LvIA selectively binds α3β2-nAChR, MII and PeIA bind α3β2- and/or α6α3β2β2-nAChRs with approximately equal affinity, and TxIB which is selective for only α6α3β2β2-nAChR.

To determine the specific amino acids of the α6-subunit that α-CTx PeIA interacts with, three residues of the α6-subunit were mutated to corresponding non-homologous residues of the α3-subunit. These mutations were E152K, D184E, and T195Q, and resulted in a >2000-fold reduced potency for the α-CTx PeIA [A7V, S9H, V10A, N11R, E14A] analog compared to α6β2β3-nAChR. An interesting feature of these mutagenesis analyses was implication of the importance of the positively charged amino acid in position 11 toward selectivity for the α6-subunit. Computational analysis using a homology model of PeIA bound to rα6 and rα3 subunits (based on the crystal structure of AChBP complexed with PnIA [A10L, D14K] (PDB: 2BR8)) indicates that this positive charge interacts with the negatively charged Glu-152 of the α6-subunit. This residue is Lys152 in the α3-subunit, and the E152K mutation significantly reduced potency for α-CTx PeIA [N11R]. This finding is corroborated by α6β2β3-nAChR specificity observed in α-CTx TxIB, in which the amino acid in position 11 is lysine. It may be concluded that α-CTxs with positively charged amino acids in the 11th position, such as α-CTx TxIB or α-CTx PeIA [N11R], interact favorably with the Glu152 of the α6-subunit while binding is disfavored to α3-containing nAChRs due to a repulsive charge-charge interaction with Lys152. Furthermore, the negative charge of Asp11 of α-CTx LvIA and its corresponding interaction with Lys152 has been speculated to be important for α3β2 selectivity [55]. This adds to evidence of this residue as being a key contributor to α-CTx selectivity and potency. Figure 2 shows a structural comparison of select α-CTxs LvIA, MII, PeIA and TxIB that bind α3β2 and/or α6α3β2β2 nAChR isoforms.

In both the α3 and α6 subunits, receptor residues 184 and 195 are located in the C-loop of strands 9 and 10 (see Figure 1b,d), respectively, but do not appear to interact directly with PeIA because their side chains are oriented away from it. This C-loop is proposed to act like a hinge allowing it to move toward the complementary subunit upon α-CTx binding. Residues 184 and 195 are smaller (Asp184 and Thr195) in α6 and bulkier (Glu184 and Gln195) in α3. This may allow the C-loop of α6 to be more flexible and allow more α-CTxs to be well-accepted in the binding pocket, while causing the C-loop of α3 to be more rigid and hinder α-CTx binding.

Luo et al. proposed computational receptor-toxin binding models for α-CTx LvIA bound to rα3β2 and rα3β4 [55]. The docking trials were conducted using a homology model of Vc1.1 in complex with the rα9α10 nAChR (originally created by Yu et al. 2013 [93]) as a template. Their model shows that the negatively charged Asp11 is buried in a cluster of the charged receptor residues Asp151, Lys154, and Glu194 in α3, and Lys78 and Arg80 in β2. However, when LvIA binds to the β4 subunit, a salt bridge between Lys58 and Glu35 becomes buried from the solvent. Luo et al. suggest this desolvation energy cost may account for the lower binding of LvIA with rα3β4 compared to rα3β2.

### 4.3. α-CTx ArIB

Native α-CTx ArIB, DECCSNPACRVNNPHVCRRR, was originally identified from the genomic DNA of *Conus arenatus* and exhibited IC_50_ values for rα7 > rα6/α3β2β3 > rα3β2, of 1.81, 6.45, and 60 nM, respectively [73]. Since the peptide has high affinity for all three isoforms, ArIB mutants were designed with the intention of enhanced selectivity for rα7. Because the [A10L] mutation in PnIA shifts selectivity to rα7 over rα3β2 [88], it was reasoned that the [V11L] mutation in the homologous position of ArIB would improve selectivity for rα7 and reduce activity for rα3β2. Indeed, the α-CTx ArIB [V11L] mutation increased selectivity for rα7 relative to rα3β2, yielding IC_50_ values of 0.539 nM and 38.8 nM, respectively. Secondly, because the [L15A] mutation of MII weakens potency for rα3β2, a [V16D] mutation in the homologous position of ArIB was expected to reduce selectivity for rα3β2 [35,94]. Indeed, potency for rα3β2 (IC_50_ >10 μM) was lowered without significantly lowering the potency for rα7 (IC_50_ 1.09 nM).

### 4.4. α-CTx ImI

α-CTx ImI, with sequence GCCSDPRCAWRC, has 12 amino acids and contains a 4/3 *m* to *n* loop spacing pattern. α-CTx ImI was isolated from the crude venom of the worm-hunting *Conus imperialis* and was shown to inhibit heterologously expressed rat neuronal α7 and α9 homopentametic nAChRs with IC_50_ values of 220 nM and 1.8 µM, respectively [74,95]. Low affinity activity toward the mouse muscle type nAChR receptor α1β1γδ was also observed, with an IC_50_ of 51 µM. Subsequent pharmacological characterization reported an IC_50_ of 595 nM toward heterologously expressed hα7 nAChR, and 40.8 nM toward hα3β2 nAChRs [75]. Activity towards hα3β2 nAChRs was surprising, because no effect was observed towards rα3β2 nAChRs at 5 µM [95]. Various mutagenesis experiments to probe the structure-activity relationship between ImI potency and selectivity toward particular nAChR subtypes have been performed [76,96,97,98]. The Asp5, Pro6, and Arg7 present in the *m*-loop, as well as Trp10 in the *n*-loop were demonstrated to be critical for binding to a human α7/5HT_3_ chimera expressed in HEK293 cells [75]. Replacement of the DPR motif in the *m*-loop resulted in a two to three order of magnitude decrease in activity. The ImI [W10T] mutant demonstrated a 30-fold loss of activity, whereas maintenance of the aromatic side chain in the [W10F] mutant resulted in a modest three-fold decrease of affinity. α-CTx ImI [R7L] and [D5N] mutations resulted in two orders of magnitude diminished activity towards hα7 expressed in *Xenopus* oocytes [96]. Further investigation into the role of the aromatic side chain in position 10 demonstrated that the analogue [W10Y] maintained activity toward rat α7 expressed in *Xenopus* oocytes, indicating that substitution of Trp10 for other aromatic residues does not strongly influence the activity of ImI [97]. To probe the interaction between Pro6 and a hydrophobic binding pocket observed in co-crystallization structures of α-CTxs in complex with AChBP, analogues of α-CTx ImI containing non-natural Pro6 derivatives were synthesized and pharmacologically characterized [98]. Activity was evaluated in tsA-201 cells transiently expressing α7/5-HT_3_ chimera using a [^3^H]-methyllycaconitine completion binding assay, HEK293 cells stably expressing rα4β2, rα3β4, or rα4β4 nAChRs using a [^3^H]-epibatidine competition binding assay, and GH3 cells stably expressing hα7 nAChR using the Fluo-4/Ca^2+^ assay. Addition of polar and charged groups on Pro6, such as 4-amino, 4-guanidino, and 4-betainamidyl, resulted in no significant binding or antagonistic activity toward hα7 nAChRs. Most aromatic and hydrophobic substituents to Pro6, such as 3-phenyl, 4-phenyl, 4-benzyl, and 4-naphthylmethyl resulted in varying degrees of diminished binding to hα7 nAChRs. The loss of activity depended upon both the position and stereochemistry of the substitution. For example, the 4-(R)-phenyl analogue resulted in an ~sixfold reduction in binding, while the 4-(S)-phenyl showed no significant binding at 100 µM. However, the 5-(R)-phenyl substitution resulted in significantly enhanced binding and antagonistic activity toward hα7 nAChR compared to the native conotoxin, with an observed 10-fold increased affinity compared to the wild type. None of the analogues demonstrated significant binding to hα4β2 or hα4β4 nAChR subtypes at 100 µM. 

Computational chemistry was used to elucidate the mechanism behind the enhanced affinity of α-CTx ImI [P6/5-(R)-phenyl] substituent toward hα7 nAChR. A homology model of the hα7 nAChR ligand binding domain (LBD) was generated using Modeller 9 version 3 [99], based on the crystal structures of mouse LBD of α1 nAChR (PDB: 2QC1) [47] subunit and *Ac*-AChBP in complex with α-CTx ImI as templates (PDB: 2BYP) [44]. Docking of α-CTx ImI [P6/5-(R)-phenyl] to the hα7 nAChR LBD model was performed using Schrödinger Glide [100]. Results of computational modeling and docking experiments suggest that the observed enhanced affinity may be due to π-stacking interactions between the P6/5-(R)-phenyl and Tyr93 in the receptor. This study was inspired by crystallographic data of α-CTxs in complex with AChBPs and demonstrates the utility of rational mutagenesis of α-CTxs to enhance binding affinity. Results of this rational design method demonstrate the utility of computational chemistry to broaden molecular insights of structure-activity relationships for the interaction of mutated CTxs with target receptors. 

In contrast to the rational design approach focused on chemical modification of a single amino acid described in the previous study, α-CTx ImI has also been used as a template in an expansive positional scanning synthetic combinatorial library (PS-SCL) experiment [101]. This experiment acquired information about three amino acids mutated in the *n*-loop of the α-CTx ImI framework, with the goal of generating α-CTx ImI analogues with improved activity and selectivity for the hα7 nAChR [102]. Using this approach, the amino acids Ala9, Try10, and Arg11 were mutated to any of 22 natural and non-natural amino acids using the “tea bag” synthetic strategy. The PS-SCL resulted in a total of 10,648 combinations, which were screened for activity in GH3 cells stably expressing hα7 nAChR using the Fluo-4/Ca^2+^ assay. In the initial screening process, 66 PS-SCL mixtures, each containing 484 unique compounds, were evaluated to determine the amino acid at a given position that resulted in the greatest potency for that mixture and were selected as promising candidates for more potent analogs. From this initial screen, the most active amino acids at position 9 were determined to be norvaline (F), with Leu and Ile at this position resulting in mixtures with high antagonist potency. In position 10, Trp was determined to be most potent, with Phe and Tyr also resulting in potent mixtures. In position 11, substitution with His proved most impactful, with Trp and α-aminobutyric acid (Abu) also resulting in potent mixtures. Thirty-six individual analogs were synthesized with position 9 occupied by Phe, Leu, or Ala (native), position 10 occupied by Trp, Phe, or Tyr (native), and position 11 occupied with Abu, His, Trp, or Arg (native). Sixty additional individual analogs were synthesized resulting in a subsequent screening of 96 compounds. The result of this study was creation of a α-CTx ImI mutant with 14-fold greater potency than native peptide; the mutant peptide contained Leu9, 4-aminophenylalanine (Aph) in position 10, and Abu11. The consensus sequence of the most potent mixtures from the initial screen –Nva9, Trp10, His11– had nearly a nine-fold enhanced potency and was one of the most effective analogs tested for inhibition of hα7 nAChR. A key observation from this study is that no single substitution resulted in increased antagonist potency, but rather, multiple substitutions acted synergistically to achieve significant increases in antagonist potency. This implies that although native toxins are well optimized for their interactions with the receptors, antagonist activity can be significantly enhanced by substitution with combinations of two or three residues.

### 4.5. α-CTx BuIA

**Figure 3:**
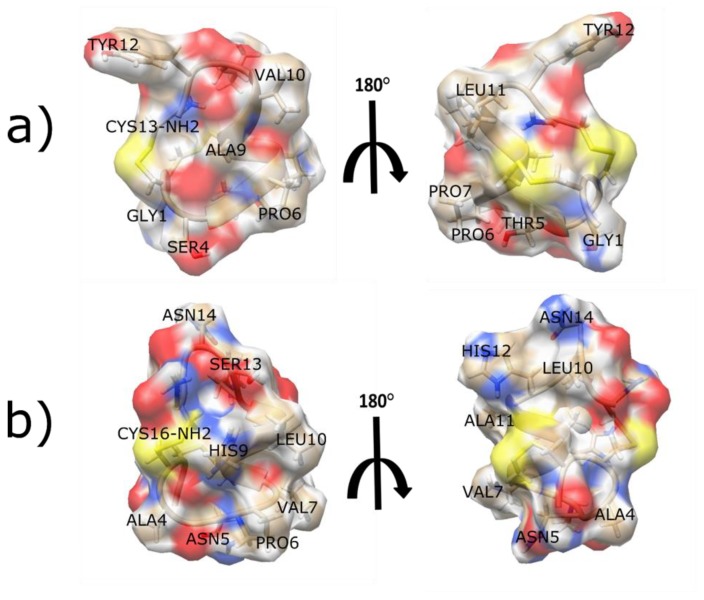
Differences in electrostatics of two conotoxins (**a**) α-CTx BuIA and (**b**) α-CTx MII [S4A, E11A, L15A] that exhibit different selectivity. The increased positive charge, contributed by Asn14, His12 and His9, on the α-CTx MII [S4A, E11A, L15A] as compared to α-CTx BuIA the is thought to contribute to the different selectivity profile of these peptides (see Table 1). The negative charge contributed by Tyr12, Thr5 and Ser4 of α-CTx BuIA is thought to contribute to binding affinity for α6α3β4-nAChR.

α-CTx BuIA, with sequence GCCSTPPCAVLYC, was originally discovered by reverse transcription of RNA isolated from the venom duct of *Conus ballatus*. This peptide has a 4/4 *m*/*n* loop framework and can block multiple rat nAChR isoforms with nanomolar potency [77]. Chemical synthesis of the mature toxin allowed for pharmacological characterization through electrophysiology experiments in *Xenopus* oocytes expressing multiple rat nAChRs. Inhibition of several different nAChR subtypes was demonstrated, with the greatest potency shown for α6/α3β2 (IC_50_ 0.258 nM), α6/α3β4 (IC_50_ 1.54 nM), α3β2 (IC_50_ 5.72 nM), and finally α3β4 (IC_50_ 27.7 nM). Notably, it was discovered through kinetic studies that α-CTx BuIA can differentiate between rβ2 versus rβ4 containing nAChRs. It was shown that the blockage of rβ2-containing nAChRs is quickly reversible (<10 min), while blockage of rβ4 containing nAChRs is slowly reversible (>25 min). With this kinetic knowledge, researchers designed mutant analogs of α-CTx BuIA to determine if selectivity with respect to potency, not just k_off_, could be achieved. This led to the creation of the α-CTx BuIA [T5A, P6O] double mutant, which is ~200 fold more selective for the rα6/α3β4 nAChR (IC_50_ 58.1 nM) as compared to rα6/α3β2β3 nAChR, (IC_50_ > 10,000 nM) [78]. Figure 3 shows the differences in electrostatic surface between α-CTx MII [S4A, E11A, L15A], an analog of α-CTx MII that is highly selective for α6/α3β2β3-nAChR isoform discussed below [90], and α-CTx BuIA [T5A, P6O] that exhibit different selectivity for the rα6/α3β2β3-nAChR isoform.

To understand how α-CTx BuIA differentiates between nAChR isoforms, another study mutated receptor residues 185-205 of the rα4 subunit to corresponding residues in rα6 subunit [103]. More specifically, mutation of three rα4 residues—Y185, T187, R188—to the corresponding rα6 residues (K185, T187, and I188) showed ~2000-fold increase in α-CTx BuIA potency. 

To determine the mechanism underlying this observation, molecular modeling was used to build a model of BuIA bound to rα4-α6(185-188)-α4β2 extracellular domain using the structure of the *Torpedo marmorata* AChR (PDB: 2BG9) and BuIA/AChBP co-crystal structure (PDB: 4EZ1) as the templates. While the potency profile implicates these residues, the molecular modeling experiment showed α-CTx BuIA binding more than 10 Å away from K185, D187, and I188. It was hypothesized that these critical three residues of the α6 subunit do not interact with BuIA directly, but instead potentially alter the structure and flexibility of the C-loop, which in turn may account for heightened potency. Figure 3 shows the sequence alignment of human α3, α4 and α6 subunits with conserved residues highlighted.

In order to enhance selectivity and potency toward inhibition of α3β4 nAChRs, α-CTx BuIA has been used as the framework for a positional-scanning synthetic combinatorial library (PS-SCL) experiment by Chang et al. [79]. α3β4 nAChRs have been implicated in nicotine addiction and drug abuse, and potent and selective antagonists of these receptors may help elucidate the role of the receptors in these conditions [104,105]. Chang et al. replaced six residues in the primary sequence of α-CTx BuIA with 22 natural and non-natural amino acids, while conserving the four cysteine residues, Gly1, Ser4, and Pro6. In total, 113,379,904 sequences were generated using the “tea bag” method with solid phase peptide synthesis (SPPS) [101]. The initial screening was performed on 132 PS-SCL mixtures, each containing 5,153,632 compounds using the fluorescent membrane potential assay in HEK293 cells stably expressing either rat α3β4, α3β2, or α7 nAChRs.

A second generation of analogues was chosen based upon the two amino acid residues identified in each position that exhibited the greatest inhibition of α3β4 nAChR subtypes. The second-generation library consisted of 64 sequences, and screening was again conducted using the fluorescent membrane potential assay in α3β4 nAChRs expressing HEK293 cells. Eleven sequences exhibited greater than 80% inhibition compared to 10 μM mecamylamine, which was the control treatment defined as 100% inhibition. Four compounds were further analyzed by competitive inhibition of [^3^H]-epibatidine with α3β4 nAChR and α4β2 nAChR expressing HEK293 cells, with no significant binding to α4β2 nAChR observed. Ultimately, this experiment resulted in the discovery of the novel mutant peptide, named TP-2212-59, with the sequence α-CTx BuIA [T5H, P7B, A9F, V10B, L11Z], where B is 2-aminobutyric acid and Z is norvaline. Functional characterization of TP-2212-59 was undertaken using electrophysiology in *Xenopus* oocytes heterologously expressing α3β4, α3β2, or α7 nAChRs. The approach taken by researchers to mutate this non-selective conotoxin to selectively bind the target receptor, resulted in ~10-fold improvement in IC_50_ from 27.7 nM to 2.3 nM in α3β4 nAChRs. No inhibition was observed for α3β2 and α7 nAChRs at 10 μM, meaning a >1,000-fold selectivity for the α3β4 nAChR subtype had been established. 

### 4.6. α-CTx Lt1.3

Recently, Chen, et al. identified and synthesized α-CTx Lt1.3, GCCSHPACSGNNPYFC [80]. They characterized the peptide with rα3β2 nAChR and found it to have an IC_50_ of 44.8 nM. As previously stated, smaller hydrophobic residues at position 10 in α-CTxs have been found to increase selectivity for rα3β2 nAChRs. It follows that Lt1.3 has Gly in position 10, and consequently selectively targets the rα3β2 nAChR isoform. The α-CTx Lt1.3 [S9A] mutant exhibits slightly enhanced potency, while [Y14A] has decreased potency by nearly five-fold. Given this level of nAChR activity adjustment, with minor primary sequence alteration, future characterization of this peptide is anticipated.

### 4.7. α-CTx TxIB

Native α-CTx TxIB, with sequence GCCSDPPCRNKHPDLC, is selective for rα6/α3β2β3 nAChRs (IC_50_ 28 nM) [81]. This peptide demonstrated little or no inhibition of other tested nAChR subtypes at concentrations up to 10 μM. The [K11A] mutant of α-CTx TxIB exhibits selectivity for rα7 nAChRs with an IC_50_ of 200 nM [82]. While most α-CTxs have a hydrophobic patch in loop 1, the hydrophobic patch in TxIB, composed of only the four amino acids SDPP, is comparatively smaller. TxIB has two patches of positive charge character on either side of its hydrophobic patch. When rβ4 rather than rβ2 was co-expressed with α6/α3, inhibition by TxIB dropped by 400-fold, indicating that slight residue differences between homologous β2 and β4 subunits significantly influence binding [82]. Similarly, when α3 rather than α6/α3 was co-expressed with β2, antagonism by TxIB was 400-fold lower, indicating that slight residue differences between homologous α6 and α3 nAChR subunits also significantly influence binding.

Yu et al. studied TxIB binding with hα7 and rα7 [82]. The ligand binding domain sequence of rα7 differs from the ligand binding domain sequence of hα7 by only 10 residues. The α-CTx TxIB [K11A] mutation (in loop 2) improved the potency towards rα7, but not hα7. Computer models of toxins complexed with a hα7-AChBP chimera indicate the importance of Arg185 to this result: receptor mutation R185K drastically decreased the activity of α-CTx TxIB [K11A] to in inhibit rα7, whereas the inverse R185K mutation in hα7 essentially conferred onto hα7 the affinity level of rα7. The importance of R185 has also been implicated in modeling studies of PnIA [A10L, D14K] and ImI with hα7. 

### 4.8. α-CTx GIC

α-CTx GIC, GCCSHPACAGNNQHIC, has a nearly identical sequence to Lt1.3 [S9A], differing by only the last four C-terminal residues [83]. α-CTx GIC inhibits hα3β2, hα4β2 and hα3β4 nAChRs, with IC_50_ values of 1.1 nM, 309 nM, and 755 nM, respectively. As with TxIB, the hydrophobic patch in GIC is comparatively smaller than most α-CTxs, suggesting GIC may have high selectivity for hα3β2. Indeed, GIC has remarkably high potency for hα3β2 nAChR isoforms (IC_50_ ~1 nM), compared to its next closest rival α-CTx MII, which has been the model antagonist for this receptor with an IC_50_ of 0.5 nM [92]. GIC and MII share identical *m*-loops but differ in seven out of eight residues in the *n*-loop, such that GIC is significantly more hydrophilic than MII.

Lin et al. built a model of the rα3β2 nAChR with bound GIC, based on the crystal structure of GIC bound to *Ac*-AChBP as a template (PDB: 5CO5), and proposed the key binding interactions between ligand and receptor [84]. Compared with TxIA [A10L] and ImI, GIC does not contain the same Arg5 or Trp10 residues conferring rα3β2 nAChR selectivity, but rather His5 (on the principal side of the receptor binding interface) and Gln13 (on the complementary side) in the homologous positions. According to this model, the residues in GIC that are most likely to interact with rα3β2 are His5, Ala7, Asn11, Asn12, and Gln13. An alanine scan was performed on various residues (H5A, A7G, A7L, N11A, N12A, and Q13A) that concluded all mutations except Q13A decreased activity with rα3β2. The model showed Gln13 of GIC surrounded by Glu61, Val111, Ser113, Ser117 and Phe119 of the rβ2 subunit. The corresponding residues in rβ4 are Glu62, Ile113, Arg115, Ser119 and Leu121; these are all the same or similar residues as those found in rβ2 except for Arg115. It is suspected that the long side chain of Arg115 may decrease the activity with GIC in the rα3β4 nAChR subtype due to steric hindrance with Gln13 in GIC. Other interactions identified by the authors were between Asn11 of GIC and Asp152 in the rα3 subunit, and between His5 of GIC and three tyrosine residues (Tyr93, Tyr190 and Tyr197) in the rα3 subunit. Indeed, α-CTx GIC [H5A] showed decreased activity with rα3β2, likely from an absence of the tyrosine interactions.

### 4.9. α-CTx TxID

α-CTx TxID (GCCSPHVCSAMSPIC), is a α-4/6-CTx identified from the genomic DNA of *Conus textile* that inhibits rα3β4, rα6/α3β4 and rα2β4 with IC_50_ values of 12.5 nM, 94 nM and 4.5 μM, respectively [85]. A series of activity assessment experiments for TxID were performed, similar to those described for TxIB [80]. By replacing rβ2 for rβ4 during co-expression with rα3, it was found that TxID potency was >800-fold lower, indicating that slight residue differences between highly homologous β2 and β4 subunits significantly influence binding, although the specific receptor residues involved in binding remain to be elucidated. Similarly, when α4 rather than α3 was co-expressed with β4, potency was >1000-fold lower, again indicating that amino acid residue differences between homologous α3 and α4 (for example, residues 148 and 196) significantly influence binding. TxID has a three amino-acid sequence of SHP in loop 1, which is present in several other α-CTxs with differing nAChR selectivity. Conversely, loop 2 of TxID contains the five amino-acid sequence SAMSP, which is not found in other pharmacologically tested α-CTxs and may be important for the unique selectivity and potency for rα3β4 nAChR exhibited by TxID.

Wu, et al. performed an alanine screen to evaluate analog selectivity for rα3β4 and rα6/α3β4 and found that α-CTx TxID [S9A] had the greatest selectivity for rα3β4 as compared to rα6/α3β4 [57]. They found that S9A did not inhibit a range of other nAChR subtypes at a threshold of 10 μM, including α7, α9α10, α1β1δε, α2β2, α2β4, α3β2, α4β2, and α4β4. α-CTx TxID [S9A] appears to have a more tightly coiled α-helical between P6 and A10 as compared to native TxID. By contrast to Ser9, the hydroxyl group of Ser4 is not involved in any perceived interaction with the rα3β4 binding site, and S4A mutation was predicted to have no impact, which was consistent with experimental IC_50_ measurement of 10.8 nM compared to 3.6 nM for native TxID. Another mutation, α-CTx TxID [M11I], achieved preferential binding for rα6/α3β4 over rα3β4. According to the model produced by Wu et al. of TxID in the binding pocket of the rα3β4 and rα6β4 nAChR interfaces (a homology model based on the crystal structure of AChBP bound to a TxIA variant, and the crystal structure of hα9 [PDB: 2UZ6]), Met11 contacts Cys218 of the α3 C-loop and [M11A] would be expected to result in a change of binding mode, due to the steric hindrance induced by the larger Met11 side chain is relieved. By contrast, M11I would not be expected to create a significant steric clash with α6 in the rα6β4 model, suggesting that this mutation would not be detrimental to rα6β4 binding. Indeed, α-CTx TxID [M11I] resulted in 20-fold decreased potency with rα3β4 but did not reduce inhibition of rα6/α3β4.

Yu, et al. took previous α-CTx TxID studies further by mutating Ser9 with a series of 14 different amino acids [58]. The α-CTx TxID [S9K] mutant exhibited the greatest selectivity for rα3β4 over rα6/α3β4 of all the variations tested. The ligand binding domains of rα3β4 and rα6β4 with native TxID differ by only α subunit positions 148 and 196, where 196 is a bulkier Gln in α3 and a less bulky Thr in α6. The bulkier Gln196 interacts with the β8 strand of the α3 subunit, “pushing” the C-loop and TxID further toward the bottom of the orthosteric binding site than in the TxID−rα6β4 model, resulting in a shift of the location of Ser9 in TxID to be within the binding site. In the TxID−rα3β4 model, the Ser9 side chain does not contact the β-strands of the β4 subunit, and the binding cavity is partly solvated, suggesting Ser9 substitution with an Ala or a long side chain amino acid, such as Lys or Arg, should be well accepted. Yu, et al.’s mutation data indeed show that Ser9 substitutions with Ala, Lys, or Arg are innocuous to rα3β4 binding. The Ser9 side chain of TxID in the TxID−rα6β4 model faces a positively charged Lys81 in β4 and is also in close proximity to a negatively charged Glu58 in β4. Substitution of Ser9 with a charged residue is expected to result in charge repulsion with either Glu58 or Lys81 of the β4-subunit. Additionally, in the TxID−rα6β4 nAChR complex, the Ser9 side chain of TxID establishes a hydrogen bond with the β4 Lys81 side chain. Indeed, Ser9 mutation to a charged residue such as Asp, Lys, or Glu shows a >300-fold decrease in rα6β4 activity in all three cases. 

### 4.10. α-CTx GID

Native α-CTx GID, IRDγCCSPNACRVNNOHVC, was originally isolated by assay-directed fractionation from the crude venom *Conus geographus* [86]. The primary sequence is notable for the four-residue extended N-terminus, the post-translationally modified residues γ-carboxyglutamic acid (γ) and hydroxyproline (O), and because GID does not contain the amidated C-terminus typical of most α-CTxs. α-CTx GID was shown to inhibit rat α3β2, α7, and α4β2, with IC_50_ values of 3.1 nM, 4.5 nM and 152 nM, respectively. Deletion of the extended N-terminus affected activity at α4β2 (IC_50_ 670 nM) but did not significantly decrease activity at α3β2 (IC_50_ 4.6 nM) and α7 (IC_50_ 5.5 nM). To determine the residues that are important for α-CTx GID inhibition of rat α3β2, α4β2, and α7 nAChRs, alanine-scanning mutagenesis has been employed [106]. Asp3, Arg12, and Asn14 were determined to be critical for inhibition of α7, while Pro9 was essential for α3β2 nAChR inhibition. More recently, Banerjee, et al. measured α-CTx GID antagonist activity of rα3β2 and rα4β2 nAChRs, with IC_50_ values of 36 and 4800 nM, respectively using fluorescent membrane potential assays in HEK293 cells expressing rat nAChRs [53]. With the goal of obtaining further insights into α-CTx GID/nAChR interactions that could lead to the design of GID analogues with improved affinity for α4β2 nAChRs, a homology model of GID bound to hα4β2 was constructed. The model was created using an X-ray co-crystal structure of a PnIA variant bound to *Ac*-AChBP as a template (PDB: 2BR8). Banerjee’s models show that Asp3, Arg12, and Asn14 in α-CTx GID are required for binding the hα4β2 nAChR. Close proximity between the side chains of Arg2 and E189, the side chain of Asp3 and the backbone NH atom of Y188, and the side chains of Glu4 and R186 and K143 were observed. Banerjee synthesized mutants of these key charged GID residues to either a smaller, less bulky, side chain or a neutral side chain, both to abolish the respective receptor-peptide interaction. Each mutation resulted in loss of activity for hα4β2 and/or hα3β2, indicating the importance of these interactions in receptor binding. The negatively charged carboxylate side chain of GID Glu4 is suspected to form salt bridges with the positively charged R186 and K143 residues in the α4 binding pocket. The α-CTx GID [E4Q] mutation introduced electrostatic repulsion between the amide hydrogen atoms of the Gln4 side chain and the positively charged R186 and K143 side chains. Additionally, Ser7 is in proximity for formation of a hydrogen bond between its side chain hydroxyl group and both the S36 hydroxyl and D169 carboxyl groups of the β2-subunit, explaining the decreased potency of α-CTx GID [S7A] with hα4β2.

Steric hindrance frequently overwhelmed many of the hypotheses made by Banerjee, et al. For example, Banerjee’s model with hα4β2 also shows Val13 of GID in proximity to a cluster of aromatic residues consisting of F104 in the β2-subunit and W118 and W147 in α4-subunit, but mutation to aromatic residues decreased activity with hα4β2, presumably due to unfavorable steric interactions. Additionally, the Ala10 side chain of GID is oriented toward a polar pocket in the hα4β2 in such a way that mutation of Ala10 to serine or threonine may establish an interaction with the backbone carbonyl of S146 in α4, but the α-CTx GID [A10T] mutation resulted in loss of activity for both hα4β2 and hα3β2, which is attributed to steric hindrance of the β-methyl group of Thr10. Conversely, the α-CTx GID [A10S] mutation had comparable potency with hα4β2 and a 10-fold decrease in potency with hα3β2, indicating position 10 may still play an important role in determining the selectivity of GID between hα4β2 and hα3β2. Banerjee reported other important interactions including a polar contact between Asn15 and Y195 in α4, and a salt bridge between Arg12 and E59 in β2, suggesting α-CTx GID [R12A] would eliminate a key binding interaction. Indeed, this is observed experimentally, resulting in a >three-fold increase in the IC_50_ for rα3β2, an almost 10-fold increase in the IC_50_ for rα7, and a loss of activity with rα4β2 [106].

The α-CTx GID [V18N] mutation exhibited comparable activity with rα4β2 (IC_50_ 1.85 µM) to the native peptide (IC_50_ 4.8 uM), while the [V18Q] mutation eliminated inhibitory activity for both rα4β2 and rα3β2. Banerjee et al. explains this result by suggesting the longer glutamine side chain experiences greater steric hindrance upon binding as compared to the shorter side chain of asparagine. Important to note is that the GID [V18N] mutation exhibited no activity with rα3β2, indicating position 18 is essential for determining selectivity between rα4β2 and rα3β2. α-CTx GID [V18N] is the most selective α-CTx for rα4β2 over rα3β2 identified to date, with an IC_50_ of 1.85 µM and >100 µM, respectively. Banerjee et al.’s hα4β2 model rationalizes the observed selectivity for rα4β2, showing a potential hydrogen bond interaction between the Asn18 amide in GID [V18N] and the hydroxyl group of Y195, which is not present in hα3β2. This interaction seems to shift the location of the C-loop in hα4β2 compared to hα3β2. 

α-CTx GID inhibition of α4β2 nAChR was recently used for the development and validation of a docking algorithm integrated into ToxDock [87]. ToxDock uses ensemble-docking and conformational sampling to dock α-CTxs to nAChR homology models. The objectives of this study were to determine if an algorithm could perform two functions: (1) accurately dock α-CTx GID to the α4β2 nAChR by accounting for the conformational flexibility of these complexes, and (2) discover new α-CTx GID derivatives with functionally enhanced affinity or selectivity for α4β2 nAChRs. Three bioactive GID mutants (A10V, V13I, and V13Y), predicted by virtual screening using ToxDock, were experimentally validated. ToxDock employs two existing protocols in the Rosetta macromolecular suite [107], Rosetta FastRelax and Rosetta FlexPepDock [108,109], to provide extensive conformational sampling of both the nAChR homology model and the conotoxin to refine models of these complexes. The α4β2 nAChR extracellular domain dimer was generated using Modeller version 9.11 based upon the X-ray structures of AChBP from *A. californica* in complex with the α-CTxs PnIA [A10L, D14K] (PDB: 2BR8), ImI (PDBs: 2C9T, 2BYP), BuIA (PDB: 4EZ1), and TxIA [A10L] (PDB: 2UZ6) [44,45,110,111,112]. Docking and virtual screening of α-CTx GID and its analogs were based upon the NMR structure of α-CTx GID (PDB: 1MTQ) [106]. To generate α-CTx GID analogs, non-conserved, hydrophobic, and buried GID residues were identified as candidates for mutation. The authors identified two residues, Ala10 and Val13 of α-CTx GID as promising candidates for point mutations. Based upon these two candidate residues, a library of 256 GID point mutants was prepared in silico using PyRosetta [113]. ToxDock was used to refine the α-CTx/α4β2 nAChR complexes. Twenty-five percent of the GID mutants were predicted to be active at the α4β2 nAChR, and the four mutants, [A10V], [A10Q], [V13I] and [V13Y] were selected for synthesis and experimental characterization. The α-CTx GID [A10Q] mutant was predicted to be inactive, while the other mutants were predicted to be active at the α4β2 nAChR subtypes. Pharmacological characterization was performed using the fluorescent membrane potential assays in HEK cells stably expressing human α7, α3β2, α3β4 or α4β2 nAChR subtypes. Electrophysiology was also conducted in *Xenopus* oocytes expressing human α4β2 nAChR subtypes. Two of the three α-CTx GID analogs identified by the virtual screening, GID [A10V] and GID [V13Y], had reduced activity at the human α7 nAChR without diminished activity at the α4β2 nAChR. The IC_50_ of α-CTx GID [A10V] and α-CTx GID [V13Y] were measured at 30 μM and 3 μM, respectively, for the α4β2 nAChR subtypes, while the wild type IC_50_ was demonstrated to be 3 μM. However, the IC_50_ for the α7 nAChR were observed to be diminished, with GID [A10V] measured at >10 μM, and α-CTx GID [V13Y] measured at 4 μM, compared to wild type IC_50_ of 0.1 μM. Activity was maintained in the α-CTx GID [V13I] mutant at both receptor subtypes, with IC_50_ values of 8 μM and 0.2 μM, for the α4β2 and α7 nAChR subtypes, respectively. Potency was maintained at the α3β2 nAChR subtype for all three active mutants, with IC_50_ ranging from 1-3 nM, which is slightly more potent than the wild type, measured at 10 nM. This study demonstrated the feasibility of a predictive computational screening and experimental validation for the identification of novel α-CTx mutants with enhanced selectivity for a nAChR subtype. Further application of this approach could be used for additional peptide toxins and their ion channel targets. 

### 4.11. α-CTx PnIA

α-CTx PnIA is a 16 amino-acid peptide originally isolated from the venom of *Conus pennaceus* [114]. Pharmacological characterization of α-CTx PnIA in *Xenopus* oocytes expressing rα3β2 and rα7 nAChRs demonstrated IC_50_ of 9.56 and 252 nM, respectively. The single mutation α-CTx PnIA [A10L] resulted in an altered receptor affinity with IC_50_ values of 12.6 nM for rα7 and 99.3 nM for rα3β2 [88]. The finding that the subtle change in the hydrophobic side chain at position 10, from Ala to Leu, significantly altered receptor affinity was corroborated by additional studies [115,116]. Modification to the structural characteristics at the Ala10 position through the incorporation of natural and non-natural amino acids, focusing on linear and branched aliphatic or aromatic residues provided detailed insights to the distinctions between the binding pockets of α3β2 and α7 nAChRs [89]. These receptor differences were further explored using electrophysiology measurements in *Xenopus* oocytes expressing either rat or human α3β2 and α7 nAChRs, and by evaluating affinity for α7 nAChRs using rat brain homogenate and measuring displacement of ^125^I-α-bungarotoxin, which is known to bind selectively to the α7 nAChR subtype [117]. Polar and aromatic substitutions at Ala10 residue were found to result in a large reduction in affinity for both receptor subtypes. Molecular docking using the program AutoDock indicated hydrophobic interactions with several α7 and α3β2 receptor residues via a hydrophobic funnel that is capable of accommodating linear and branched side chains, but not aromatic chains. Extending the Ala10 to norleucine (Nle) resulted in an increased binding affinity at both α7 and α3β2 receptor subtypes, with observed IC_50_ values of 4.3 nM and 0.7 nM, respectively. The α-CTx PnIA [A10Nle] mutation reverted selectivity back to the α3β2 nAChR. The binding affinity of the A10Nle was determined to be 44 nM as determined by ^125^I-α-bungarotoxin radioligand competition studies of rat brain homogenate. Further lengthening the Ala10 side chain beyond the Nle mutant through the incorporation of amino heptanoic acid (Aha) or amino octanoic acid, extensions of one or two carbon-carbon bond lengths, respectively, resulted in decreased affinity for the α7 nAChR isoform of 136 nM and 756 nM, respectively. This study demonstrated that a single mutation to α-CTx PnIA [A10L] flips its selectivity profile from the α3β2 nAChR to the α7 nAChR, and showed the placement of a single methyl group can drastically alter binding affinity of the toxin. The impact of this finding is valuable detailed structure information about the α7 nAChR binding pocket, which may inform the development of more selective ligands in the future. This study also demonstrated the utility of computational experiments in understanding the molecular basis for ligand selectivity between α7 and α3β2 nAChRs, where the residue located at the equivalent position to R208 in α7 (equivalent to I186 in α3) is one of the key determinants for ligand selectivity.

α-CTx PnIA was recently used as a starting point to design mutants with enhanced affinity for α7 nAChR using protein surface topography (PST) [118]. PST is a computational method used to understand ligand/receptor interactions that addresses bioactive peptides and their targets as interacting surfaces [119]. Ligand surfaces are simplified and transformed into a machine-tractable format of 2D projection maps, which enable group analysis and produce a pattern that defines activity/selectivity for a group of molecules. The initial application of the PST approach was on neurotoxic peptides from scorpion venom [120]. The extension of PST to α-CTxs inhibiting the α7 nAChR benefits from the considerable number of 4/7 α-CTxs identified and structurally characterized that are available to be used for structure-function analyses aimed at mapping ligand pharmacophores. The PST computational strategy began by establishing a database of 39 α-CTxs known to block α7 nAChRs and dividing this database into three groups based upon inhibition activity: “good” (IC_50_ < 16 nM), “average” (IC_50_ 39-390 nM), and “bad” (IC_50_ > 390 nM). A structural database was generated for the 39 α-CTxs either obtained from the PDB or built using homology modeling, and 2D spherical maps were generated of hydrophobic and electrostatic properties distributed over the peptide surface using PST [118]. Analysis of the relationship between the α-CTxs’ activity and properties visualized in the 2D spherical maps were correlated, and group-averaged maps for the “good” and “bad” groups were generated. Differential maps were constructed to emphasize the prominent differences between “good” and “bad” groups, and these results were used to inform the design of α-CTx PnIA variants. Three α-CTx PnIA analogs were synthesized: PnIA [A9R], PnIA [A9R, A10L], and PnIA [L5R, A9R, A10L, D14R]. The L5R, A9R, and D14R mutations were chosen because of the observation that “good” 2D spherical maps contained more positive electrostatic potential compared to “bad” maps, and A10L mutation was chosen due to literature president for increased affinity for the α7 nAChR [88,115,116]. The three PnIA analogs were synthesized and pharmacologically characterized using radioligand analysis and electrophysiology. The competitive radioligand assay with [^125^I]-labeled α-Bgt and [^125^I]-labeled PnIA analogs were conducted in GH4C1 cells transfected with human α7 nAChR, and electrophysiology was conducted using two-electrode voltage clamp in *Xenopus* oocytes following rat α7 nAChR cDNA injection. The IC_50_ values for the three mutants were ~20 nM each, demonstrating high affinity binding to the α7 nAChR isoform. However, native α-CTx PnIA was not evaluated in this study, so a direct effect of these mutations on the potency of PnIA cannot be concluded. This study demonstrated that the PST approach can be applied to α-CTxs and may provide another computational strategy to design highly active and selective compounds for other nAChR subtypes. 

### 4.12. α-CTx MII

**Figure 4:**
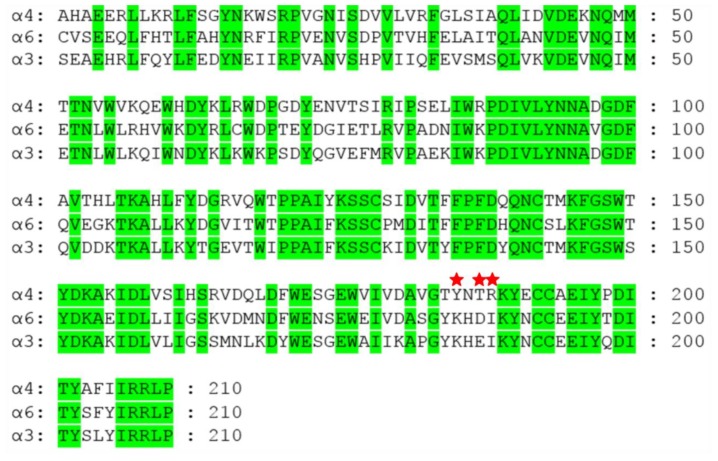
α-Subunit sequence alignment for human nAChRs; α4 and α6 have 60.00% sequence homology, α4 and α3 have 61.43% sequence homology, and the α6 and α3 have 66.67% sequence homology. Homologous residues are highlighted in green. Stars are placed above residues that were mutated to assess the influence of residues of the α4 versus α6 responsible for the selectivity of α-CTx BuIA.

α-CTx MII, originally isolated from the venom of *Conus magus*, is a 16 amino-acid peptide with the sequence GCCSNPVCHLEHSNLC [92]. α-CTx MII has a reported IC_50_ for the α3β2 nAChR isoform of 0.5 nM as determined by electrophysiology voltage clamp experiments in *Xenopus* oocytes expressing rat α3β2 nAChRs. Alanine-screening of α-CTx MII identified three residues, Asn5, Pro6, and His12, as key to α-CTx MII’s potency, with diminished activity of >2,700-fold, 700-fold, and >2,700-fold, respectively [94]. Shortly after this high affinity for α3β2 nAChRs was reported, the single site mutant, α-CTx MII [E11A] was shown to exhibit preferential binding for α6/α3β2β3 nAChR isoforms compared to α3β2 nAChR isoforms, with IC_50_s of 0.160 nM and 8.72 nM, respectively, with an observed preference for α6/α3β2β3 of >50-fold [34,35,121]. The double mutant α-CTx MII [H9A, L15A], was found to be even more selective for α6/α3β2β3 versus α3β2, with IC_50_ values of 2.40 nM and 4850 nM, respectively, a 2020-fold preference for α6/α3β2β3 nAChRs. α-CTx MII [E9A, L15A] also demonstrated ~100-fold preference for the α6/α3β2β3 versus α6/α3β4 nAChRs, with an observed IC_50_ for the later of 269 nM. Through mutagenesis experiments of both the α-CTx MII ligand and the α3β2 nAChR isoform, the residues that play a critical role in CTx ligand binding to the α6 containing nAChRs were speculated to be E152, D184, K185, I188, and T195 [90]. The α-CTx MII triple mutant, α-CTx MII [S4A, E11A, L15A] has been shown to block a chimeric receptor α6/α3β2β3 nAChR (IC_50_ = 1.2 nM) with threefold less potency than native α-CTx MII (IC_50_ = 0.39 nM), but also blocked the α3β2 nAChR isoform with >600-fold lower affinity (IC_50_ = 1400 nM) than native α-CTx MII (IC_50_ = 2.18 nM). These combined alanine substitutions resulted in mutant with >1000-fold preference for α6/α3β2β3 vs the α3β2 nAChR isoform. The potency of α-CTx MII to intact pentameric transmembrane spanning α3β2 nAChRs has been determined to require interaction with K185 and I188 residues on the α3 subunit of the receptor [122]. These two residues are present in both α6 and α3 subunit proteins, necessitating additional investigation into root determinants of α-CTx differentiation between the α3 and α6-containing nAChR isoforms. Figure 4 shows the sequence alignment of α3, α4, and α6 subunits with conserved residues highlighted in green for human nAChR proteins. Through mutating site-specific residues of the α3 subunit into the counterpart residues of the α6, it has been shown that E152, D184, and T195 play a critical role in ligand differentiation between α3β2 and α6β2 nAChR isoforms [78]. The triple mutant, α-CTx MII [S4A, E11A, L15A], showed ~150-fold increase in affinity to the α3 [K152E, E184D, Q195T] triple mutant as compared to the native α3 receptor (IC_50_s of 9.7 nM and 1400 nM, respectively). The three order of magnitude preference for α6/α3β2β3 vs the α3β2 displayed by α-CTx MII [S4A, E11A, L15A] resulted in a CTx that can clearly differentiate between these closely related nAChR subtypes.

A subsequent study aimed to alter α-CTx MII to achieved higher affinity and specificity for α6β2 containing nAChR subtypes by identifying amino acid residues in the primary sequence of α-CTx PIA (RDPCCSNPVCTVHNPQIC) that could alter the binding profile of MII [123]. α-CTx PIA was cloned from DNA isolated from the hepatopancreas of *Conus purpurascens* using PCR primers for the 3’ end of the intron preceding the toxin region of the toxin prepropeptides and the 3’ untranslated region sequence of the α-prepropeptides [124]. α-CTx PIA was the first CTx found to discriminate between α6-containing and α3-containing nAChRs, with IC_50_ values for rα6/α3β2β3, hα6/α3β2β3, rα6/α3β2 and rα3β2 of 0.95 nM, 1.72 nM, 0.69 nM, and 74.2 nM, respectively, as determined by electrophysiology in *Xenopus* oocytes heterogously expressing nAChRs. α-CTx PIA has an N-terminal RDP sequence, and to determine if this extended N-terminus is important in α6β2 selectivity, the hybrid peptide RDP-MII was synthesized. Additionally, docking studies suggested the importance of Glu11 in MII that may determine α6β2-containing vs α3β2-containing nAChR selectivity. Both the α-CTx MII [E11R] and RDP-MII [E11R] were synthesized, and their binding affinity was evaluated in native rat α6β2-containing nAChRs isolated from rat brain striatum, and in native rat α3β2-containing nAChRs isolated from rat brain superior colliculus, and measured by displacement of [^125^I]-epibatidine. K_i_ for native α-CTx MII was determined to be 5.6 nM and 62 nM for α6β2-containing and α3β2-containing nAChRs, respectively. The RDP-MII hybrid peptide demonstrated a 13-fold increased affinity for α6β2-containing nAChRs (K_i_ 0.43 nM), but similar affinity for α3β2-containing nAChRs (K_i_ 50 nM). The α-CTx MII [E11R] mutation did not alter affinity for α6β2-containing nAChRs (K_i_ 50 nM), but drastically reduced binding affinity for α3β2-containing nAChRs (K_i_ 4230 nM). The RDP-MII [E11R] resulted in enhanced binding to α6β2-containing nAChRs, with a K_i_ of 0.9 nM, and drastically reduced affinity α3β2-containing nAChRs, with a K_i_ of 4410 nM. The results of this study were discovery of the novel RDP-MII [E11R] and MII [E11R] analogs selective for α6β2-containing nAChRs, and demonstration of the utility of CTx mutagenesis based on the detailed examination of the primary sequence of other CTxs. 

**Figure 5:**
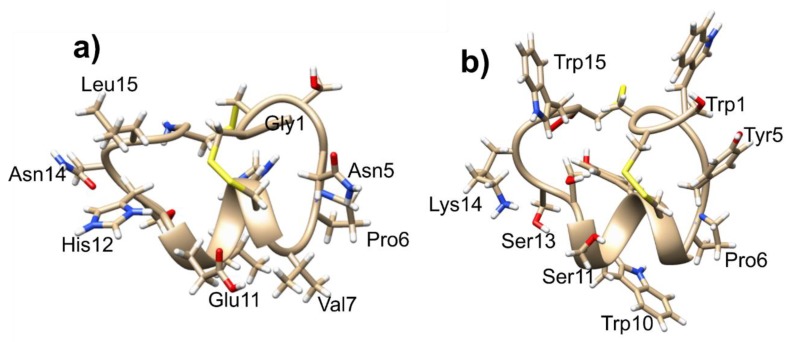
Structural comparison between (**a**) α-CTx MII and (**b**) the predicted structure of KTM. Select amino acids are labeled. The backbone scaffold is predicted to be maintained by conserving the structurally important cysteine and proline residues.

Our lab has developed an algorithm to search exceptionally large mutant libraries for sequences with optimal binding affinities for ion channel receptors [125]. The Genetic Algorithm Managed Peptide Mutant Screening (GAMPMS) program was used to search an α-CTx MII mutant library composed of approximately 41 billion possible peptide sequences for those peptides exhibiting the greatest binding affinity for the α3β2 nAChR subtype. GAMPMS is a genetic algorithm designed for comprehensive structure-based high throughput virtual screening of very large mutant libraries [9]. Genetic algorithms have found utility in computational and combinatorial chemistry applications [126,127,128,129]. GAMPMS uses the popular AutoDock 4.0 molecular docking software to provide fitness scores and uses the results of previous docking jobs to make an informed decision as to which mutations increase or decrease peptide binding affinity [130,131]. The use of AutoDock 4.0 additionally provides the added advantage of making the molecular docking results reproducible by other researchers. GAMPMS and the additional ancillary programs have been integrated into the open source DockoMatic software package, making this methodology readily available to the research community [132]. The α-CTx MII sequence was used as the base sequence for mutation, with Cys2, Cys3, Pro6, Cys8, and Cys16 residues being conserved during these simulations because of their importance in maintaining the molecular scaffold of the native peptide. The remaining 11 residues in α-CTx MII were constrained to mutations that maintained the polar/nonpolar character at each residue. Therefore, Ser4, Asn5, His9, Glu11, His12, Ser13, and Asn14, were mutated into any polar or charged amino acid excluding cysteine, and nonpolar residues Gly1, Val7, Leu19 and Leu15 residues were mutated to any nonpolar amino acids excluding proline. The result of these mutations was a large library consisting of 40.96 billion possible peptide sequences. Three-dimensional peptide mutant structures are generated by copying the coordinates of the mutable residue and two adjacent residues into a new pdb file, removing the side chain atoms of the mutable residue from the generated tripeptide pdb file, adding the substituted side chain atoms, submitting the peptide analogue to TreePack, and grafting the modified peptide segment back into the original ligand pdb file. TreePack determines the appropriate orientation of the new side chain to eliminate side-chain spatial overlaps [133]. The fitness of an individual sequence is determined by the AutoDock score produced when the ligand is docked against the target receptor. The receptor structure used in the docking calculations was a homology model of the α3β2-nAChR isoform constructed from the amino acid sequences of the α3 and β2 subunits of rat neuronal nAChR using the *Torpedo marmorata* nAChR X-ray structure (PDB: 2BG9) as a structural template [42]. The homology models were created using the DockoMatic 2.1 and MODELLER packages [99,132]. In the GAMPMS workflow, three genetic operators derived from the natural evolutionary processes of elitism, crossover, and mutation are used in determining peptide sequences with the highest “fitness,” which is binding affinity in this context. In determination of binding affinity, forty pose evaluations were used in the AutoDock docking simulation for ligand/receptor binding. Because of uncertainty in values of predicted binding free energies from molecular docking scoring functions, the best indicator that a side-chain mutation resulted in a beneficial effect on the predicted binding affinity is not the docking score directly but rather the conservation of any particular mutation that is observed among a population exhibiting the best docking scores. The conservation of residues across the peptides with the highest binding affinities was used to determine the final top sequence. The residue occurring with the highest frequency in the top 50 peptides, with all residues having a conservation of at least 50%, was included in the consensus sequence: WCCSYPGCYWSSSKWC. This consensus sequence, given the trivial name KTM, was subjected to further investigation and validation using molecular dynamics (MD) simulations and compared to sequences with known binding affinities with the α3β2 nAChR isoform. MD simulations were performed using the GROMACS 5.0.4 software package with the AMBER03 force-field parameter set [134,135,136,137,138,139]. The GAMPMS-derived molecular docking results were used as the input structures for the MD simulations. The calculated ΔG_bind_ for KTM was −45.59 kcal/mol, which is more than double that of MII at a predicted ΔG_bind_ of −20.42 kcal/mol. The binding free energies of the reference PnIA and TxIA peptides were −31.53 kcal/mol and −32.20 kcal/mol, respectively. Although the calculated binding free energies of PnIA and TxIA are difficult to correlate with that of MII in absolute terms since there is no direct translation between ΔG_bind_ and experimentally obtained IC_50_ values, the relative ΔG_bind_ is in agreement with experimental results showing the significant predicted enhancement in binding affinity of these peptides for the α3β2 nAChR [111]. Figure 5 shows a comparison between the 3D structures of α-CTx MII and KTM. 

This study used the GAMPMS protocol to produce an α-CTx MII analogue with computationally predicted optimal binding to the α3β2 nAChR by heuristically searching the set of all possible amino acid residue combinations. The resulting consensus mutant peptide, KTM, was shown to have considerably higher binding affinity for the α3β2 nAChR than the precursor α-CTx MII peptide. However, the assumption that the allowed mutations would not alter the secondary structure of the α-CTx MII template is dubious, and the correlation between binding affinity and IC_50_ requires experimental validation to confirm channel pore influence. The KTM peptide has been synthesized and work is currently underway in our lab to determine the 3D structure of this peptide to validate the veracity of the predicted structure, as well as to experimentally test its pharmacological properties.

## 5. Conclusions

The work described herein exemplifies the value of conotoxins as molecular probes for understanding the delicate intricacies of nAChR isoform selectivity and inhibition of channel pore proteins required to develop treatments for neurological disorders. Despite the large number of α-CTxs that have been characterized, either through isolation from *Conus* venom, or synthesis based on sequences identified in *Conus* transcriptomes, designing potent and selective ligands for a particular nAChR subtype is still a challenge. The purpose of this review was to summarize strategies undertaken by researchers to alter the primary sequences of α-CTxs to enhance their affinity and selectivity for particular nAChR subtypes. Numerous examples of alanine scanning mutagenesis were illustrated. Mutagenesis inspired by detailed comparison of the primary sequence of several CTxs was illustrated in the examples of PeIA [72] and the MII [123]. Mutation of multiple amino acids through positional scanning synthetic combinatorial libraries was illustrated for ImI [102] and BuIA [79]. Particular attention was paid to novel computational strategies that hold promise in limiting the cost and time required to develop more potent and selective α-CTx analogs, such as the novel ToxDock program used to introduce mutations to GID [87], the Protein Surface Topography approach used for PnIA [118], and the Genetic Algorithm Managed Peptide Mutant Screening algorithm in DockoMatic used for extensive mutation of MII [125]. Collectively, the work summarized in this review demonstrates the unique value provided by conotoxins to enhance our understanding of complex molecular systems. 

## Figures and Tables

**Table 1:** CTxs and analogs discussed in the text, with primary sequence and nAChR isoform target shown. Mutations in analog CTxs are shown in red. Non-natural amino acids are indicated as follows: B: 2-aminobutyric acid; Z: norvaline; O: hydroxyproline; γ: γ-carboxyglutamic acid; P/5(R)-Ph: proline-5-(R)-phenyl; Aph: 4-aminophenylalanine; Nle: norleucine. IC_50_ values were determined by electrophysiology, unless otherwise noted.

α-CTx	Primary Sequence	nAChR Isoform (IC_50_)	Ref.
Vc1.1	GCCSDPRCNYDHPEIC	rα3β4(4.2 μM) > rα3α5β2(7.2 μM) > rα3β2(7.3 μM)	[67]
		rα9α10(109 nM) > hα9α10(549 nM) > rα3β2(5.5 μM)	[70]
	GCCSDPRCAYDHPEIC	rα9α10(13 nM) > hα9α10(27 nM) > rα3β2(185 nM)	
	GCCKDPRCAYDHPEIC	hα9α10(19 nM) > rα9α10(93 nM) > rα3β2(>3 μM)	
PeIA	GCCSHPACSVNHPELC*	rα9α10 (6.9 nM) > rα6/α3β2β3 (17.2 nM) > rα3β2 (19.2 nM) > rα3β4 (480 nM)	[71]
	GCCSHPVCHARHPALC*	rα6/α3β2β3 (2.16 nM) > rα3β2 (30.9 μM)	[72]
ArIB	DECCSNPACRVNNPHVCRRR*	rα7 (1.81 nM) > rα6/α3β2β3 (6.45 nM) > rα3β2 (60.1 nM)	[73]
	DECCSNPACRLNNPHACRRR*	rα7 (0.356 nM) > rα3β2 (74.5 nM) > rα6/α3β2β3 (120 nM)	
	DECCSNPACRLNNPHDCRRR*	rα7 (1.09 nM) > rα6/α3β2β3 (828 nM) > rα3β2 (>10 μM)	
ImI	GCCSDPRCAWRC*	rα7 (220 nM) > rα7 (1.8 μM) > mα1β1γδ (51 μM) hα3β2(40.8 nM) > hα7 (595 nM)	[74] [75]
	GCCSDP/5(R)-PhRCAWRC*	hα7 (0.70 μM)|native (2.6 μM) > rα3β4 (3.7 μM) | native (>300 μM)	[76] ^δ^
BuIA	GCCSTPPCAVLYC*	rα6/α3β2 (0.258 nM) > rα6/α3β4 (1.54 nM) > rα3β2 (5.72 nM) > rα3β4 (27.7 nM)	[77]
	GCCSAOPCAVLYC*	rα6/α3β4 (58.1 nM) > rα3β4 (1.2 μM) > rα6/α3β2β3 (>10 μM)	[78]
	GCCSHPBCFBZYC*	α3β4 (2.3 nM) > α3β2 (>10 μM) ≈ α7 (>10 μM)	[79]
Lt1.3	GCCSHPACSGNNPYFC*	rα3β2 (44.8 nM)	[80]
	GCCSHPACAGNNPYFC*	rα3β2 (35.4 nM)	
	GCCSHPACSGNNPAFC*	rα3β2 (216 nM)	
TxIB	GCCSDPPCRNKHPDLC*	rα6/α3β2β3 (28 nM)	[81]
	GCCSDPPCRNAHPDLC*	rα7 (200 nM) > hα7 (>10 μM)	[82]
GIC	GCCSHPACAGNNQHIC*	hα3β2 (1.1 nM) > hα4β2 (309 nM) > hα3β4 (755 nM)	[83]
	GCCSHPACAGNNAHIC*	hα3β2 (8.41 nM) > hα3β4 (660 nM)	[84]
TxID	GCCSPHVCSAMSPIC*	rα3β4 (12.5 nM) > rα6/α3β4 (94 nM) > rα3β4 (4.5μM rα3β4 (3.6 nM) > rα6/α3β4 (34 nM)	[85]
			[58]
	GCCSPHVCAAMSPIC*	rα3β4 (3.9 nM) > rα6/α3β4 (178 nM)	[57]
	GCCSPHVCRAMSPIC*	rα3β4 (5.4 nM) > rα6/α3β4 (350 nM)	[58]
	GCCSPHVCDAMSPIC*	rα3β4 (380 nM) > rα6/α3β4 (>10 μM)	[58]
	GCCSPHVCSAISPIC*	rα6/α3β4 (50 nM) > rα3β4 (75 nM)	[57]
	GCCSPHVCKAMSPIC*	rα3β4 (6.9 nM) > rα6/α3β4 (>10 μM)	[58]
GID	IRDγCCSPNACRVNNOHVC	rα3β2 (3.1 nM) > rα7 (4.5 nM) > rα4β2 (152 nM)	[86]
	----CCSPNACRVNNOHVC	rα3β2 (4.6 nM) > rα7 (5.5 nM) > rα4β2 (670 nM)	
	IRDECCSPNACRVNNOHVC	rα3β2 (36 nM) > rα4β2 (4.8 μM)	[53] ^θ^
	IRDECCSPNACRVNNOHNC	rα4β2 (1.85 μM) > rα3β2 (>100 μM)	
	IRDECCSPNACRVNNOHVC	hα3β2 (10 nM) > hα7 (100 nM) > hα4β2 (3 μM)	[87] ^θ^
	IRDECCSPNACRYNNOHVC	hα3β2 (20 nM) > hα4β2 (3 μM) > hα7 (4 μM)	
PnIA	GCCSLPPCAANNPDYC-*	rα3β2 (9.56 nM) > rα7 (252 nM)	[88]
	GCCSLPPCALNNPDYC-*	rα7 (12.6 nM) > rα3β2 (99.3 nM)	
	GCCSLPPCANleNNPDYC-*	rα3β2 (0.7 nM) > rα7 (4.3 nM)	[89]
MII	GCCSNPVCHLEHSNLC-*	rα6/α3β2β3 (0.39 nM) > rα3β2 (2.18 nM)	[35]
	GCCSNPVCHLAHSNLC-*	rα6/α3β2β3 (0.16 nM) > rα3β2 (8.72 nM)	
	GCCSNPVCALAHSNAC-*	rα6/α3β2β3 (0.16 nM) > rα6/α3β4 (269 nM) > rα3β2 (4850 nM)	
	GCCANPVCHLAHSNAC-*	rα6/α3β2β3 (1.2 nM) > rα3β2 (1400 nM)	[90]

^*^ Indicates amidated C-terminus. ^δ^ Indicates that IC_50_s were determined by Fluo-4/Ca^2+^ assay. ^θ^ Indicates that IC_50_s were determined by fluorescent membrane potential assays. In the case of data generated for α-CTx ImI using the Fluo-4/Ca^2+^ assay, the IC_50_ of the native CTx is included, because of the discrepancy observed between inhibitory values determined by the Fluo-4/Ca^2+^ assay and electrophysiology measurements.
